# The Case of a 59‐Year‐Old Woman With Acute Right‐Sided Weakness and Progressive White Matter Changes

**DOI:** 10.1002/acn3.70543

**Published:** 2026-11-11

**Authors:** Yookyong Baek, Hyunah Choi, Ilena George

**Affiliations:** ^1^ Thomas Jefferson University Hospital Philadelphia Pennsylvania USA; ^2^ SUNY Downstate Medical Center Brooklyn New York USA

**Keywords:** CADASIL, leukoencephalopathy, multiple sclerosis mimic, stroke

A 59‐year‐old Chinese woman with hypertension, diabetes, lacunar stroke, and a 20‐year history of relapsing–remitting multiple sclerosis (RRMS) treated with interferon beta‐1 presented with 3 days of acute right‐sided weakness and sensory loss. She had been reportedly relapse‐free since her MS diagnosis, though serial MRIs showed progressive lacunar infarcts without new demyelinating lesions. Several months prior, she was started on aspirin and statin therapy for secondary stroke prevention.

Neurological examination revealed right‐sided pronator drift, 4/5 strength in the right arm and leg, brisk right‐sided deep tendon reflexes with an ipsilateral Babinski sign, decreased right‐sided pinprick and vibration sensation, decreased facial sensation in the V1–V3 distribution, and right leg circumduction on gait.

Brain MRI demonstrated multifocal acute infarcts involving the left pons, midbrain, left cerebral peduncle, and anterior right temporal lobe. Serial neuroimaging over the preceding two decades revealed progressive lacunar infarcts and extensive confluent T2/FLAIR hyperintensities in the periventricular and subcortical white matter, bilateral anterior temporal lobes, brainstem, and cerebellum—imaging patterns recognized as characteristic markers of CADASIL [1, 2]—along with scattered microhemorrhages in the bilateral thalami and basal ganglia [3]. Spinal cord MRI showed no demyelinating lesions. MRA demonstrated atherosclerotic stenosis of the internal carotid siphons, and TEE identified an immobile aortic arch plaque. Laboratory studies were notable for HbA1c of 11.2% and LDL of 132 mg/dL. ANA and ANCA were negative.

The distribution of white matter changes was not fully explained by atherosclerotic disease alone, prompting genetic testing of the *NOTCH3* gene [4], which revealed a heterozygous pathogenic missense mutation (c.244T>C [p.Cys82Arg]), confirming a diagnosis of CADASIL.

## 1 Diagnosis

Cerebral autosomal dominant arteriopathy with subcortical infarction and leukoencephalopathy (CADASIL).

## 2 Take‐Home Points

- Diagnosis of CADASIL can be challenging due to overlapping clinical and radiological features with other neurological conditions, particularly MS. Both CADASIL and MS may present with recurrent neurologic symptoms and share imaging findings such as periventricular, brainstem, and corpus callosum white matter changes.
- Characteristic imaging features of CADASIL can aid diagnosis. Key radiologic markers include hyperintensity of the anterior temporal poles and the external capsule–insular region (Figures 1 and 2).
- The gold standard for diagnosing CADASIL is identification of a pathogenic NOTCH3 mutation on chromosome 19, inherited in an autosomal dominant pattern.
- Although CADASIL is primarily a small vessel disease caused by degeneration of vascular smooth muscle cells, large artery abnormalities may also occur and can contribute to stroke burden, suggesting an interplay between the genetic arteriopathy and traditional atherosclerotic processes.

**FIGURE 1:**
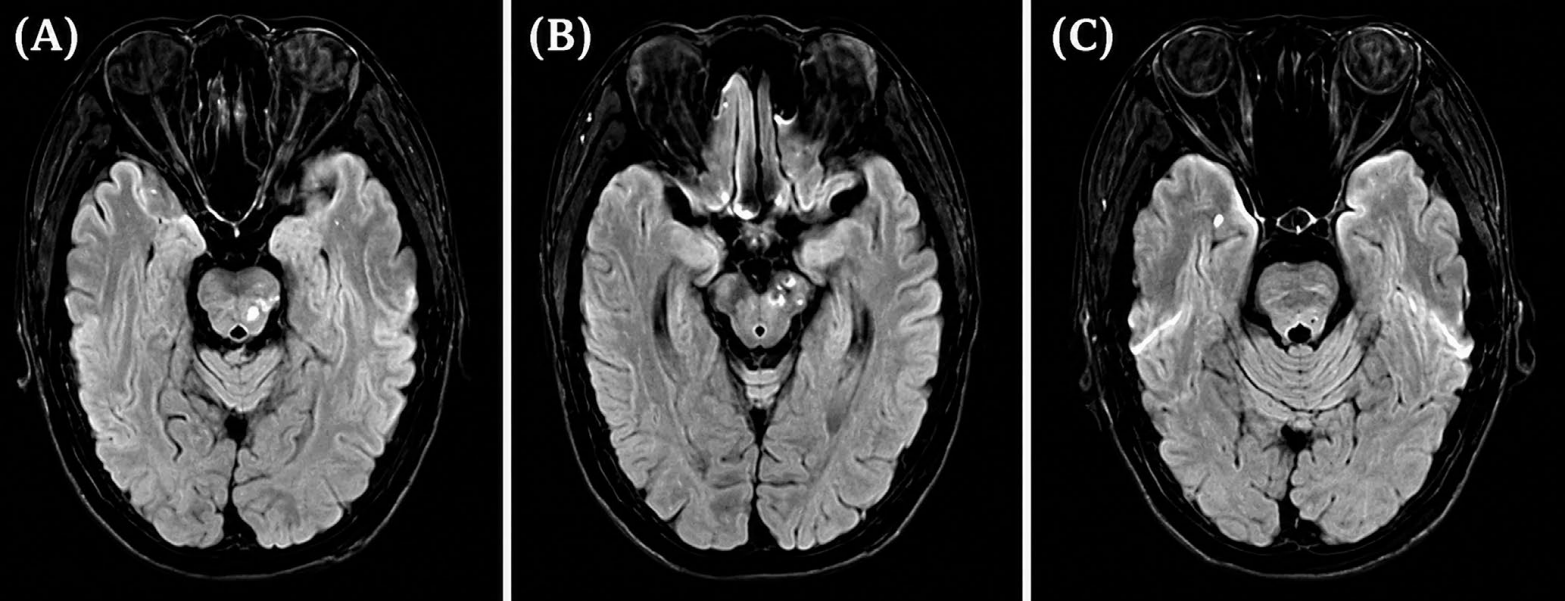
Diffusion‐weighted imaging (DWI) brain MRI demonstrating hyperintensity in the left paramedian pons extending into the midbrain and the left cerebral peduncle (A, B), and a small focus of restricted diffusion in the anterior right temporal lobe (C).

**FIGURE 2:**
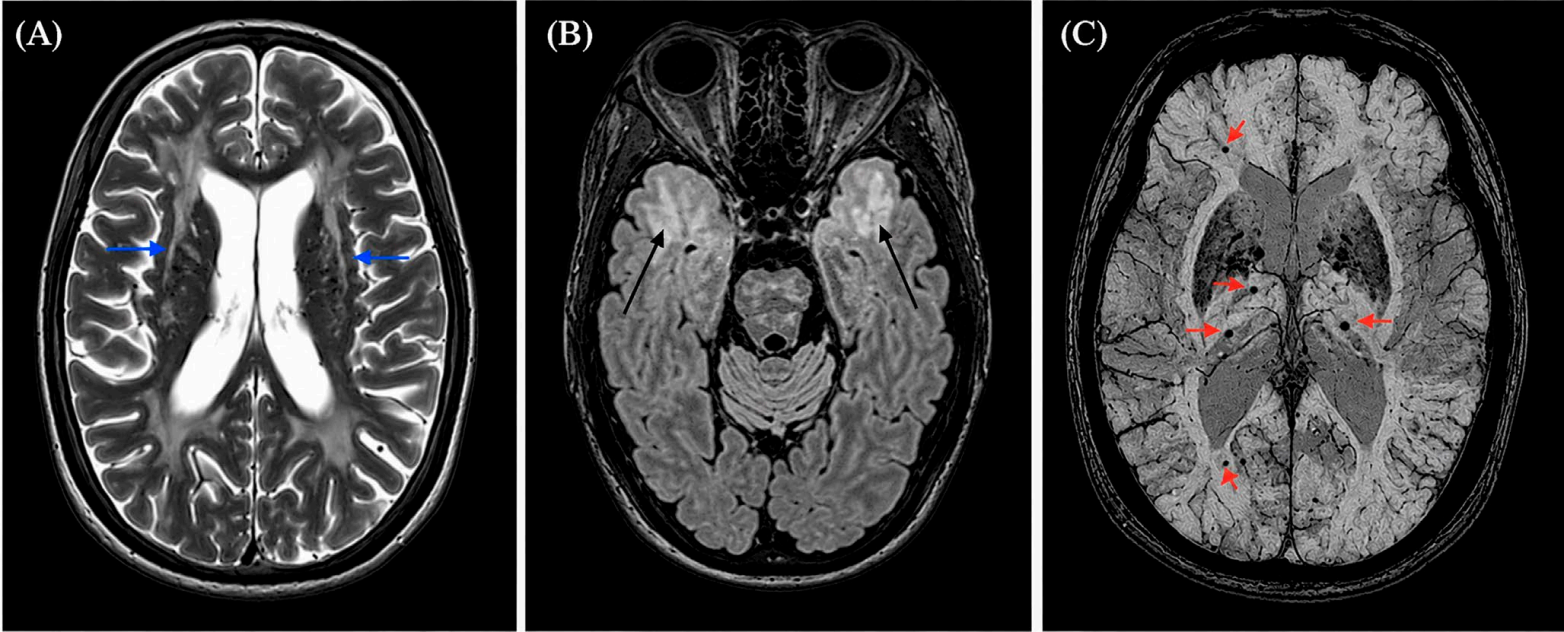
(A) Axial T2‐weighted MRI demonstrating extensive confluent periventricular and subcortical white matter hyperintensities with extension into the bilateral external capsules (blue arrows). Several chronic lacunar infarcts are also present. (B) Axial 3D SPACE T2/FLAIR reconstruction showing bilateral confluent subcortical hyperintensities involving the anterior temporal lobes (black arrows). (C) Maximum intensity projection (MIP) image from susceptibility‐weighted imaging (SWI) demonstrating scattered microhemorrhages in the bilateral thalami and in subcortical regions (red arrows).

## Author Contributions

**Yookyong Baek:** conceptualization, investigation, writing – original draft, writing – review and editing. **Hyunah Choi:** investigation, writing – review and editing. **Ilena George:** conceptualization, supervision, writing – review and editing.

## Funding

The authors have nothing to report.

## Conflicts of Interest

Dr. Ilena George is an employee and stockholder of Bristol Myers Squibb. This work is outside the scope of that employment. The other authors declare no conflicts of interest.

## Data Availability

The data that support the findings of this study are available from the corresponding author upon reasonable request.
